# Tumour budding: a promising parameter in colorectal cancer

**DOI:** 10.1038/bjc.2012.127

**Published:** 2012-04-24

**Authors:** A Lugli, E Karamitopoulou, I Zlobec

**Affiliations:** 1 Clinical Pathology Division, Institute of Pathology, University of Bern, Murtenstrasse 31, Bern 3010, Switzerland

**Keywords:** tumour budding, colorectal cancer, prognosis, scoring system, TNM stage

## Abstract

In 2011, the Tumour Node Metastasis (TNM) staging system still remains the gold standard for stratifying colorectal cancer (CRC) patients into prognostic subgroups, and is considered a solid basis for treatment management. Nevertheless, there is still a challenge with regard to therapeutic strategy; stage II patients are not typically selected for postoperative adjuvant chemotherapy, although some stage II patients have a comparable outcome to stage III patients who, themselves do receive such treatment. Consequently, there has been an inundation of ‘prognostic biomarker’ studies aiming to improve the prognostic stratification power of the TNM staging system. Most proposed biomarkers are not implemented because of lack of reproducibility, validation and standardisation. This problem can be partially resolved by following the REMARK guidelines. In search of novel prognostic factors for patients with CRC, one might glance at a table in the book entitled ‘Prognostic Factors in Cancer’ published by the International Union against Cancer (UICC) in 2006, in which TNM stage, L and V classifications are considered ‘essential’ prognostic factors, whereas tumour grade, perineural invasion, tumour budding and tumour-border configuration among others are proposed as ‘additional’ prognostic factors. Histopathology reports normally include the ‘essential’ features and are accompanied by tumour grade, histological subtype and information on perineural invasion, but interestingly, the tumour-border configuration (i.e., growth pattern) and especially tumour budding are rarely reported. Although scoring systems such as the ‘BRE’ in breast and ‘Gleason’ in prostate cancer are solidly based on histomorphological features and used in daily practice, no such additional scoring system to complement TNM staging is available for CRC. Regardless of differences in study design and methods for tumour-budding assessment, the prognostic power of tumour budding has been confirmed by dozens of study groups worldwide, suggesting that tumour budding may be a valuable candidate for inclusion into a future prognostic scoring system for CRC. This mini-review therefore attempts to present a short and concise overview on tumour budding, including morphological, molecular and prognostic aspects underlining its inter-disciplinary relevance.

## 

The management of colorectal cancer (CRC) patients is ideally multidisciplinary and includes the fields of oncology, surgery, radio-/oncology, gastroenterology and pathology. The pathologist's main role is to provide an optimal histopathology report including all information that allows the clinician to design the best therapeutic strategy for the patient. According to the International Union against Cancer's (UICC's) publication ‘Prognostic Factors in Cancer’, Tumour Node Metastasis (TNM) stage, as well as L and V classifications are considered ‘essential’ prognostic factors, whereas tumour grade, tumour budding, tumour-border configuration, perineural invasion, medullary type, CEA and perforation are considered ‘additional’ prognostic factors (Compton, 2006). Although the diagnostic histopathology report will normally include the essential prognostic factors, there is still a challenge with regard to therapy. Stage II CRC patients are typically not selected for postoperative adjuvant chemotherapy, although some patients with stage II disease may experience an outcome similar to stage III patients who do receive an adjuvant treatment. In the last few years, the aim of many studies has been to identify biomarkers capable of stratifying stage II CRC into better prognostic subgroups. The consequence has been an inundation of analyses proposing *the* biomarker, but ultimately, because of lack of reproducibility, validation or standardisation, is rejected from implementation in some instances, perhaps prematurely. The REMARK guidelines proposed in 2005 try to improve the quality of biomarker studies by suggesting a strict study design (McShane *et al*, 2005).

An interesting observation is that in contrast to breast and prostate cancer where the ‘BRE’ and ‘Gleason’ score are used in daily routine, there has been no real advancement with respect to additional prognostic factors or scoring systems in CRC. Although tumour grade is consistently reported, it is a feature which suffers from poor interobserver reproducibility and does not have the expected impact on prognosis (Chandler and Houlston, 2008). In search for additional prognostic factors, the question arises whether it would not be advantageous to re-evaluate other simple histomorphological features such as tumour-border configuration and tumour budding and their potential clinical value.

Tumour budding reflects a detachment of tumour cells at the invasive front of CRC into single cells or clusters up to five cells (Ueno *et al*, 2002). Tumour budding is diagnosed at high magnification and should not be confounded with the tumour-border configuration (infiltrative or pushing pattern) that is more easily diagnosed on a low magnification. Indeed, the combination of these two features reflects four prognostic CRC subgroups (Figure 1A–D; Ueno *et al*, 2002). Many studies have highlighted the prognostic power of tumour budding using different cohorts, different scoring systems and methods of assessment.

The aim of this mini-review is to try to sensitise the reader to tumour budding by presenting a short and concise overview.

## Morphological and molecular aspects of tumour budding

Biologically, the aim of tumour buds seems clear, namely to fight themselves through the peritumoural connective tissue, to evade the host's defence and finally invade the lymphatic and blood vessels with the consequence of local and distant metastasis. The process of tumour budding has been linked to epithelial–mesenchymal transition (EMT), which allows a polarised cell to assume a more mesenchymal phenotype with increased migratory capacity, invasiveness, resistance to apoptosis and production of extracellular matrix (ECM) molecules (Kalluri, 2009). Although formally tumour budding has not been equated with EMT, several parallels between the two processes, including activation in WNT signalling, can be drawn (Muto *et al*, 2006).

The first step in a tumour bud's life seems to be its detachment from the main tumour body by loss of membranous expression of the adhesion molecule E-cadherin. Indeed, aggressive, dissociated tumour buds not only lose membranous E-cadherin expression (a cytoplasmic expression can still be found), but also express fibronectin within the cytoplasm, implying a more mesenchymal phenotype underlining the interaction between tumour buds and the surrounding stroma (Kirchner and Brabletz, 2000). Activation of WNT signalling is further implied by an evident expression of *β*-catenin within the nucleus rather than in cytoplasm or membrane in tumour-budding cells, as well as increased laminin 5 gamma 2 and activation of SLUG and ZEB1(Muto *et al*, 2006; Schmalhofer *et al*, 2009).

**Figure 2:**
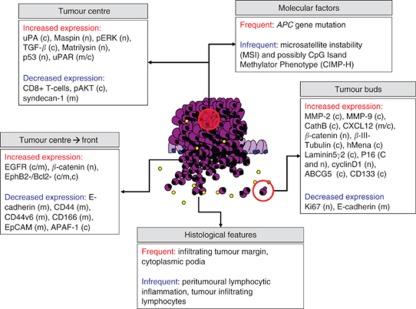
Overview of the histomorphological and molecular features of the tumour centre, invasive front and tumour buds in CRC. Reproduced from Zlobec and Lugli, 2010.

Tumour buds may have a role in ECM degradation, a hypothesis supported by increased immunohistochemical expression of proteins such as matrix metalloproteinases MMP-2 and MMP-9, and urokinase plasminogen-activator receptor (uPAR) in high-grade tumour-budding cases (Zlobec and Lugli, 2010). This process should intuitively be associated with an increased cellular proliferation, but paradoxically, the expression of the proliferation marker Ki-67 is reduced in tumour buds (Muto *et al*, 2006). Additionally, the invasion, migration, angiogenesis and chemotaxis potential of tumour buds and stem cell-like character has been shown in several studies that analysed markers such as uPAR, matrilysin, CD44, epithelial cell adhesion molecule, MMP-7 and MMP-9, *β*(III)-tubulin and CXCL12 (Zlobec and Lugli, 2010; Figure 2).

## Prognostic value of tumour budding

Over the last 5 years, the number of publications investigating tumour budding as a predictor of lymph node positivity, local and distant relapse, lymphovascular invasion and poor prognosis among patients of all pathological stages has markedly increased. In fact, tumour budding is now considered a category IIB prognostic factor (Compton, 2012). Tumour budding and lymph node metastasis are closely linked. The presence of buds is considered an independent predictor of node-positivity among patients with submucosally invasive, or early pT1-2 disease (Choi *et al*, 2009; Komori *et al*, 2010; Tateishi *et al*, 2010) and is proposed as an indicator for isolated tumour cells in lymph nodes of pN0 patients (Park *et al*, 2005; Choi *et al*, 2009; Komori *et al*, 2010; Tateishi *et al*, 2010). The frequency of tumour budding increases with more advanced TNM stage (Hase *et al*, 1993) and is a predictor of venous and lymphatic invasion as well as of distant metastasis (Nakamura *et al*, 2005; Wang *et al*, 2009) and local recurrence, even among patients with early or node-negative disease (Tanaka *et al*, 2003; Prall *et al*, 2005). Moreover, it has been suggested that tumour budding be used as a criteria to identify patients with adenoma and early T1 tumours requiring complete resection (including the regional lymph nodes) after endoscopic resection (Yasuda *et al*, 2007).

Tumour budding is a factor of poor prognosis, and in most studies, independent of pathological stage. In 1993, Hase *et al* conducted a study on 663 patients showing first that tumour budding led to worse outcome and second, that Dukes' B patients with tumour budding had similar, if not worse, survival than Dukes' C patients with no budding (Hase *et al*, 1993). This was again documented by Okuyama *et al* (2003); they report no difference in survival between node-negative patients with tumour budding and those with node-positive disease. Ueno *et al* (2004) used two large cohorts of 638 and 476 patients undergoing potentially curative surgery; tumour budding was found to be an independent prognostic factor in both. Tumour budding occurs less frequently in microsatellite-unstable high CRC compared with microsatellite-stable tumours, but has an unfavourable prognostic impact in both molecular subgroups (Jass *et al*, 2003; Zlobec *et al*, 2011). This negative effect on outcome may, however to some degree, be tempered by the presence of immune cells, in particular CD8+, FoxP3+ and CD68+ cells within the tumour-budding microenvironment, suggesting that a balance between ‘attackers’ and ‘defenders’ at the invasion front, that is, tumour buds *vs* specific immune cell types, may be more important than the contribution of either separately (Zlobec *et al*, 2011).

Although tumour budding is typically thought of as a diagnosis restricted to the invasive front of CRC, Morodomi *et al* (1989) reported the presence of tumour budding within pre-operative biopsies. This is highly relevant, as in daily practice, CRCs are almost always diagnosed by evaluating ‘superficial’ colonoscopic biopsies not encompassing the tumour invasion front. The presence of dissociated tumour cells within the main tumour body has been coined ‘intratumoural’ budding (ITB), to distinguish it from the classical ‘peritumoural’ budding’ (PTB) (Lugli *et al*, 2011). In two different patient subsets totalling more than 500 patients, we could show that ITB was not only strongly correlated with PTB, but is additionally associated with lymph node positivity, more advanced TNM stage, vascular invasion and poor patient outcome in both univariate and multivariate analyses. Most recently, Giger *et al* (2012) showed the strong specificity of ITB for PTB and lymph node metastasis in corresponding resections. Even in cases with no PTB, the presence of ITB appears to be highly specific for lymph node metastasis and linked to adverse prognosis. The clinical relevance of ITB and its potential impact on patient management are still in their early stages of investigation. However, ITB may be unique in its role as a prognostic factor, as it could relay information on outcome already in the pre-operative setting.

The growing body of evidence supports tumour budding as an aggressive and adverse prognostic factor in all pathological stages of disease (Hase *et al*, 1993; Ueno *et al*, 2004; Nakamura *et al*, 2005; Wang *et al*, 2009). Despite these encouraging data, no clinical trials have to date assessed the contribution of tumour budding in the prospective setting, and in particular its potential impact among stage II patients. Moreover, the reporting of tumour budding during daily diagnostic routine suffers from the absence of consensus over a standardised scoring system for its assessment.

## Tumour budding in daily practice

**Figure 3:**
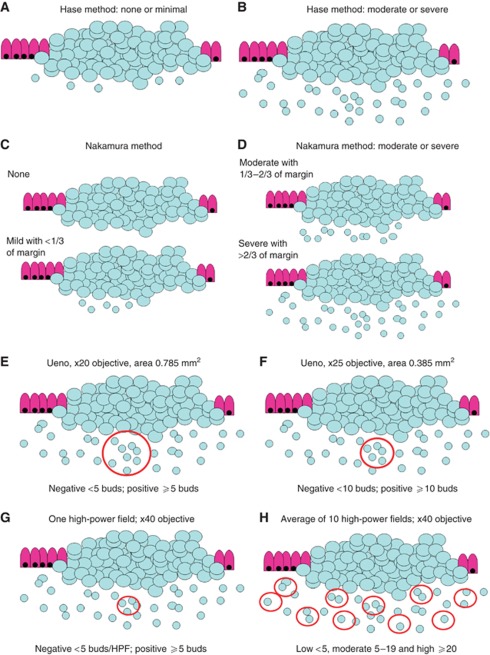
Visualisation of the proposed tumour budding scoring systems according to Hase *et al*, 1993 (**A**, **B**), Nakamura *et al*, 2005 (**C**, **D**), Ueno *et al*, 2002 (**E**, **F**), one high-power field (**G**) and 10 high-power fields (**H**) average.

In daily practice, a pathology report should include information on TNM, L and V, tumour grade and the histological subtype (Edge *et al*, 2010). Although the inter-observer variability of tumour grade is less than optimal, this feature is still commonly used and by far more frequently reported than tumour budding (Chandler and Houlston, 2008). Many studies have concluded similarly that tumour budding is a strong independent prognostic parameter using different scoring systems such as those originally proposed by Hase *et al* (1993); Ueno *et al* (2002) and Nakamura *et al* (2005) (Figure 3A–F). These findings suggest that despite the lack of a standardised scoring system, tumour budding is a reliable marker of tumour progression and bad outcome even independently of the evaluation system used. A further possibility for scoring tumour budding may be a ‘hot-spot’ approach by analysing just one high-power field (Figure 3G). The advantage of this scoring method is probably the speed with which the pathologist can report tumour budding, but the probability that different pathologists select the same ‘hot spot’ for evaluation is likely low and would lead to high interobserver variability.

On the basis of experience with other reported histomorphological features in various tumour types such as the mitoses count in breast cancer, soft tissue tumours and gastrointestinal stromal tumours, a reliable method for scoring such features is the 10 high-power fields average (Figure 3H). Indeed, this scoring system applied to tumour budding would have several advantages (Koelzer *et al*, 2011), such as ease of implementation, flexibility in selecting regions for assessment and subsequent categorisation of CRC cohorts into prognostic subgroups using, for example, either a two-tier or three-tier scoring system.

## Conclusion

In summary, tumour budding should be considered a promising and strong prognostic factor in CRC. Its definite implementation will depend on a selected, internationally accepted scoring system. Additionally, based on functional studies, tumour buds could be a potential target for new therapeutic approaches.

## Figures and Tables

**Figure 1:**
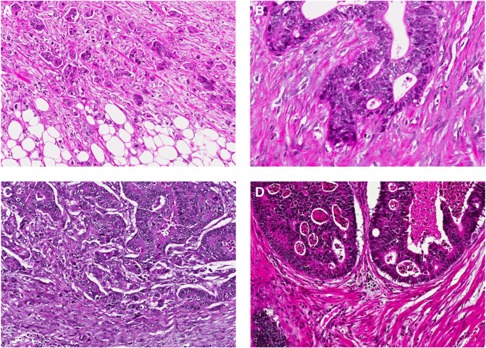
H&E stain showing the difference between the two additional prognostic parameters – tumour budding and tumour-budding configuration in CRC. (**A**) Infiltrating tumour-border configuration and presence of many tumour buds at the invasive front. (**B**) Infiltrating tumour-border configuration without tumour buds; in this case, the invasive front includes only tumour glands. (**C**) Pushing tumour-border configuration and presence of several tumour buds and (**D**) pushing tumour-border configuration without tumour budding.
